# Navigating antibiotic therapy in acute cholangitis: Best practices and new insights

**DOI:** 10.1002/jhbp.12087

**Published:** 2024-11-13

**Authors:** Sakue Masuda, Yoshinori Imamura, Ryuhei Jinushi, Karen Kimura, Shomei Ryozawa, Kazuya Koizumi

**Affiliations:** ^1^ Department of Gastroenterology, Medicine Center Shonan Kamakura General Hospital Kamakura Japan; ^2^ Cancer Care Promotion Center University of Fukui Hospital Fukui Japan; ^3^ Department of Gastroenterology Saitama Medical University International Medical Center Saitama Japan

**Keywords:** antimicrobial resistance, antimicrobial stewardship, cholangitis, duration of antimicrobial therapy, endoscopic retrograde cholangiopancreatography

## Abstract

Globally, antibiotic resistance is linked to increased morbidity, mortality, and healthcare costs, which necessitates further research on optimal antibiotic usage. Acute cholangitis (AC), a common cause of community‐acquired bacteremia, often requires antimicrobial therapy. Therefore, studying the appropriate use of antibiotics for AC is considered crucial for suppressing the emergence of resistant bacteria and reducing adverse antibiotic‐associated events. The Tokyo Guidelines 2018 (TG18) recommend 4–7 days of antibiotics post‐biliary drainage. However, this lacks strong evidence and is based primarily on various evidence and expert opinions. Recent retrospective studies advocate for a shorter 1–3‐day antibiotic course for AC, thereby prompting a need to reassess the treatment duration to balance therapeutic efficacy and minimize resistance and adverse effects. Choosing the appropriate duration and antibiotics based on susceptibility to pathogens causing cholangitis is important. Awareness of local resistance patterns and understanding patients' risks of resistant pathogens are prerequisite for effective treatment. We must explore the applicability of these guidelines in specific scenarios such as severe AC, positive blood cultures, fever, or hilar biliary obstructions due to malignancy. This comprehensive review considers both the duration and type of antibiotics and aims to enhance treatment outcomes while reducing the risk of resistant bacterial infections.

## Graphical abstract

Antibiotic resistance necessitates re‐evaluation of the Tokyo Guidelines 2018 for acute cholangitis, specifically the recommended 4–7 days of antibiotics post‐biliary drainage. Recent studies suggest that 1–3 days may suffice. This review by Masuda and colleagues addresses both duration and type of antibiotics to improve outcomes while minimizing resistant bacterial infections.

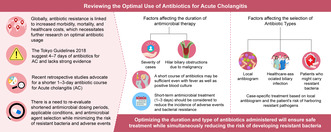

## 1 INTRODUCTION

Acute cholangitis (AC), the second or fourth most common infectious disease leading to community‐acquired bacteremia, frequently necessitates the use of antimicrobial therapy owing to its high prevalence.^1^, ^2^ The 30‐day mortality rate for acute cholangitis is reported to be approximately 1%–2%, 2%–5%, and 5%–8% in mild, moderate, and severe cases, respectively, thereby indicating that fatal outcomes have not become uncommon.^3^, ^4^

The Tokyo Guidelines 2018 (TG18), which are well‐recognized AC guidelines, state that managing this condition centers on antimicrobial therapy and biliary drainage.^5^ TG18 recommends 4–7 days as the standard duration of antibiotic administration after successful biliary drainage. However, the recommendations of TG18 for the duration of antimicrobial therapy are based on a conglomeration of diverse evidence, integrated through expert opinion, with insufficient specific evidence to support them.^5^ Recent retrospective studies suggested that a 1–3‐day course or less of antibiotic therapy following biliary drainage is a reasonable duration for antibiotic administration to cure AC.^6^, ^7^, ^8^, ^9^ Thus, most studies on the duration of antibiotic administration for AC have assumed successful biliary drainage. This is particularly critical in moderate‐to‐severe cases, where early biliary drainage is recommended to reduce mortality rates as advocated in the TG18.^10^

While evidence is accumulating for the use of antibiotic therapy in the treatment of AC, research on its adverse events is also actively being conducted. The risk of developing resistant organisms has been reported to increase with each additional day of antimicrobial therapy.^11^ Furthermore, long‐term antimicrobial administration has been reported to increase *Clostridium difficile* enteritis and invasive candidiasis, antimicrobial‐induced organ damage, healthcare costs, and mortality in case of some infections (intra‐abdominal infections).^12^, ^13^, ^14^, ^15^, ^16^, ^17^ It is necessary to determine the treatment duration that maximizes the therapeutic efficacy of antibiotics while minimizing adverse events.

The decision on antibiotic therapy to cure AC requires not only consideration of the duration of treatment, but also the type of antibiotic. It is desirable to select antibiotics that are susceptible to pathogens causing cholangitis. Therefore, it is essential to be aware of the local antibiogram^8^, ^18^, ^19^, ^20^, ^21^, ^22^ and understand patient backgrounds that are susceptible to resistant bacterial infections.^11^, ^19^, ^23^, ^24^, ^25^, ^26^, ^27^, ^28^, ^29^, ^30^, ^31^, ^32^, ^33^, ^34^, ^35^, ^36^, ^37^, ^38^, ^39^ AC is a common disease requiring antimicrobial therapy.^1^, ^2^ Therefore, studying the appropriate use of antibiotics for AC is considered highly significant for suppressing the emergence of resistant bacteria and reducing the adverse events associated with antibiotics.

As such, there has been an increase in studies focusing on the duration of antimicrobial therapy in recent years; however, since the publication of TG18, there has been limited accumulation of data based on randomized controlled trials (RCTs). However, significant progress has been made through retrospective studies, particularly in the understanding and management of special situations.^8^, ^40^, ^41^, ^42^ Therefore, our review aims to highlight and consolidate these developments in order to provide scientific value. We believe our manuscript contributes meaningfully by addressing several critical issues such as the duration of treatment, specific conditions like severe grades, positive blood cultures, fever, or multiple hilar biliary obstructions due to malignancy, and the selection of antibiotics. In this review, it was assumed that biliary drainage was technically successful for the duration of antibiotic administration.

## 2 DURATION OF ANTIMICROBIAL THERAPY FOR CURE OF AC

Standard treatment for acute cholangitis in the early 2000s typically involved the administration of antibiotics for approximately 10 days.^43^ Currently, TG18 recommends a 4–7‐day duration for the administration of antibiotics after biliary drainage. However, this recommendation has been challenged by a recent RCT conducted in India.^44^ This phase 3 trial, focusing on cases of moderate and severe AC, compared treatment durations of four and 8 days, and ultimately confirmed the noninferiority of a shorter duration. The necessary duration of antibiotic therapy for the cure of AC appears to be at the shorter end of the standard 4–7‐day period. Furthermore, recent retrospective studies suggested that a 1–3‐day course or less of antibiotic therapy following biliary drainage is a reasonable duration for antibiotic administration to cure AC.^6^, ^7^, ^8^, ^9^

When evaluating these studies, it is important to consider a few aspects. First, the outcomes differed between studies, thereby making comparisons challenging. Furthermore, previous studies have used outcomes that are not suitable for infectious diseases. Table 1 presents the representative outcomes utilized in reports until 2022.^7^, ^8^, ^9^, ^21^, ^43^, ^45^, ^46^, ^47^, ^48^, ^49^ The most common primary endpoint was recurrence of AC, but the duration varies among studies, ranging from 3 days to 4 weeks, and even up to 3 months.^7^, ^9^, ^43^, ^46^, ^47^, ^48^, ^49^ The second most common primary endpoint was 30‐day mortality; however, it has become sufficiently low in recent years, even among severe cases, therby no longer making it a point of contention. Therefore, a more appropriate selection of endpoints is desirable. To address this issue, we proposed the clinical cure rate as an outcome. Currently, the FDA recommends the clinical cure rate as an outcome in studies of urinary tract infections, which rank among the most common infectious diseases.^50^ Indeed, the previously mentioned phase 3 trial in India selected the clinical cure rate as the primary endpoint rather than focusing on the recurrence of AC or 30‐day mortality.^44^ Another issue is that many previous studies set the short‐course treatment group at 3 days or less based on the TG18 guidelines. However, the control group included patients who received treatments longer than the standard duration proposed by TG18, specifically treatments lasting 8 days or more. This grouping was inappropriate for investigating the noninferiority and adverse events of short‐course treatment compared with standard treatment.

**TABLE 1:** Representative outcomes.

	Primary outcome	Secondary outcome
Occurrence of a local infectious complication (such as liver abscess or acute cholecystitis)	2	4
Recurrence of acute cholangitis	4	3
30‐day mortality	3	3
Increased severity	1	1
Length of hospitalization	1	5
In‐hospital mortality	1	1
Adverse events of antibiotics	1	
Eradication of bacteria in the bloodstream 30 days after diagnosis of bacteremia	1	
*C. difficile* colitis		1
Normalization of blood test (such as white blood cell or c‐reactive protein)		2
Surgical intervention as an additional treatment		1

Two RCTs, BOLT‐P3 (https://jrct.niph.go.jp/en‐latest‐detail/jRCT1031230709) and COBRA (https://clinicaltrials.gov/study/NCT05750966) are currently in progress. In these trials, despite some differences, the clinical cure rate was set as the primary outcome, the control groups were aligned with the administration duration recommended by TG18, and the objective was to verify that short‐term treatment was not inferior to standard therapy. If these results are reported within 2–3 years, the duration of antibiotic administration for AC might shift towards the shorter end of the spectrum, as suggested by previous retrospective studies, thus, potentially recommending treatment periods as brief as 3 days or shorter. Table 2 summarizes the eligibility, exclusion, and primary outcomes of the two ongoing RCTs and one completed RCT in India.

**TABLE 2:** Eligibility criteria, exclusion criteria, and primary outcomes of ongoing or completed randomized controlled trials.

	BOLT‐P3 (Japan, *n* = 200)	COBRA (Netherland, *n* = 440)	Indian RCT (India, *n* = 120)
PICO	P Patients of AC who have had successful biliary drainage	P Patients of AC who have had successful biliary drainage	P Patients of AC who have had successful biliary drainage
	I 1–3‐day course of antibiotic therapy	I 1‐day course of antibiotic therapy	I 4‐day course of antibiotic therapy
	C 4–7‐day course of antibiotic therapy	C 4–7‐day course of antibiotic therapy	C 8‐day course of antibiotic therapy
	O Clinical cure rate	O Clinical cure rate	O Clinical cure rate
Eligibility criteria	1. Acute cholangitis conforming to TG18 guidelines	1. Acute cholangitis	1. Moderate to severe acute cholangitis in accordance with TG18 guidelines
	2. Benign or malignant status is not considered However, for patients with acute cholangitis due to distal biliary stent dysfunction, only those with stents placed for more than 30 days are included	2. Cholelithiasis, benign or malignant stricture of the distal biliary duct, obstruction of the distal biliary stent (only stents in place for a minimum of 30 days)	2. Patients who have had successful biliary drainage within 48 h of hospitalization
	3. Cases where the removal of biliary obstruction mechanisms was technically successful within 48 h of admission	3. Cases where sufficient biliary drainage has been successful (all common bile duct stones are removed and/or there is an adequate flow of clear bile with or without a biliary stent[s])	3. Age 18 and over
	4. Age 18 and over	4. Temperature below 38.5°C or a decrease at least once within 24 h after ERCP	
		5. Age 18 and over	
Exclusion criteria	1. Septic shock	1. Individuals currently admitted to the ICU.	1. Patients requiring mechanical ventilation
	2. Currently admitted to the ICU	2. Patients with recurrent cholangitis within 3 months	2. Patients with a Glasgow Coma Scale score of less than 8
	3. Recurrent cholangitis within the last 3 months	3. Primary sclerosing cholangitis or cancer of the biliary tract at the hepatic hilum	3. Patients suspected of incomplete biliary drainage: those with primary sclerosing cholangitis or portal biliopathy with multiple intrahepatic biliary strictures, and patients with Bismuth type III or IV strictures at the hepatic hilum
	4. Biliary obstruction of the hepatic portal region of Bismuth type II or higher	4. Patients with reconstructed intestine	4. Patients with cholangitis‐related complications that require long‐term antibiotic therapy, such as liver abscess, acute cholecystitis, or diagnosed with infectious endocarditis, pneumonia, septic arthritis, or other metastatic complications
	5. Primary sclerosing cholangitis	5. Concurrent diseases at the time of enrollment: acute pancreatitis, cholecystitis, hepatic abscess	5. Patients with acute pancreatitis and other septic foci, necessitating an extended period of antibiotic therapy
	6. Patients with reconstructed intestine	6. Co‐occurrence of another infection	6. Immunocompromised patients (those receiving chemotherapy, daily prednisolone at a dose of 10 mg, or other immunosuppressive drugs)

	7. Complications with other acute diseases such as acute pancreatitis, acute cholecystitis, or hepatic abscess	7. Regular use of antimicrobial or immunosuppressive drugs	7. Pregnant women
	8. Patients who have been receiving continuous administration of antimicrobial agents prior to enrollment	8. Neutropenia	
	9. Specific immunosuppressed states	9. Body temperature above 38.5°C or less than a 1‐degree decrease in body temperature since admission	
	10. Hypothermia with a body temperature below 35°C		
	11. Pregnant women		
12. Other cases deemed inappropriate for this trial by the attending physician		
Primary outcome	**Clinical cure rate**	**Clinical cure rate**	**Clinical cure rate**
	The proportion of cases that achieve clinical symptom improvement within 14 days, without recurrence, and survive. Clinical symptom improvement refers to the complete disappearance of pretreatment clinical symptoms (chills, fever exceeding 37.5°C, abdominal pain, jaundice). Recurrence is defined as the reappearance of any of the following in cases of cholangitis where clinical improvement had been confirmed and antimicrobial therapy had been discontinued: (1) Clinical signs suggestive of cholangitis recurrence, (2) Signs of infection in the liver, pancreas, or biliary tract areas, (3) Other signs of infection that are considered to be related to the initial episode of cholangitis, necessitating the resumption of antimicrobial therapy	Achievement of clinical symptom improvement by day 14 after ERCP, with no recurrence by day 30. Clinical symptom improvement is defined as the absence of fever exceeding 38°C and shivering, with the disappearance of symptoms present at the time of consultation. Recurrence is defined as the initiation of new antimicrobial therapy, recurrence of cholangitis, or the occurrence of subsequent infections in the hepato‐biliary‐pancreatic region or infections that are considered to be related to the cholangitis	Clinical cure is defined as the absence of cholangitis recurrence by day 30 and a 50% reduction in bilirubin levels compared to the initial value 15 days after successful biliary drainage

*Note*: PICO is a framework used in medical research, consisting of “Patient or Population,” “Intervention,” “Comparison,” and “Outcome.”

Abbreviations: AC, acute cholangitis; ERCP, endoscopic retrograde cholangiopancreatography; ICU, intensive care unit; TG18, Tokyo Guidelines 2018.

## 3 SPECIFIC CONDITIONS

The application of standard or short‐term treatment to specific conditions, such as severe grade, positive blood cultures, fever, or multiple hilar biliary obstructions due to malignancy, remains uncertain. Regardless of whether biliary drainage is performed, there is little evidence supporting the methods of antibiotic administration for cholangitis accompanied by these conditions. However, recent studies have addressed these specific conditions with successful biliary drainage,^8^, ^40^, ^41^, ^42^, ^44^ and their findings are discussed in this paper. Additionally, common complications like pyogenic liver abscesses and acute cholecystitis will also be covered.

### 3.1 Cases of severe AC

To the best of our knowledge, there is no high‐quality evidence suggesting that the duration of antimicrobial therapy should be altered based on the severity of the condition, and that the treatment duration recommended by TG18 does not differ based on severity. The Indian RCT, limited to moderate and severe cases of AC, compared antimicrobial therapy durations of 4 and 8 days after biliary drainage and reported no difference in outcomes, even in severe cases.^44^ Currently, a treatment duration of 4 days after biliary drainage seems sufficient, regardless of the severity; however, there is no high‐quality evidence for treatment durations of less than 4 days. The results of the ongoing BOLT‐P3 and COBRA studies are anticipated. However, in these three RCTs, patients requiring ICU admission due to extreme severity are excluded, and past retrospective studies have not focused on these most severe patients either.^7^, ^8^, ^9^, ^21^, ^43^, ^45^, ^46^, ^47^, ^48^, ^49^ Therefore, it would be appropriate to determine the duration of antimicrobial therapy for the most severe cases of cholangitis based on the course and consultation with intensivists of each individual case.

### 3.2 Positive blood cultures

Several retrospective studies have shown that short‐course treatments do not worsen outcomes even in AC patients with positive blood cultures.^8^, ^21^, ^40^ In response, the French Society of Infectious Diseases recommends a short course of antibiotic therapy (within 3 days), even for cholangitis with bacteremia.^51^ Although the level of evidence is low, TG18 recommends 2 weeks of antibiotic therapy for patients with gram‐positive cocci (GPC)‐positive blood cultures because of concerns about infected endocarditis (IE). Gomi et al. validated 6433 patients with cholangitis and found 243 cases with GPC‐positive blood cultures; however, no complications of IE were observed in those cases. In this study, the overall incidence of IE was 0.26% (*N* = 17 cases).^4^ Although 2 weeks of antibiotic therapy may be appropriate in patients at risk for IE, such as those with valvular disease and chronic poor oral hygiene,^52^ complications of IE are rare in patients with cholangitis. Therefore, two‐weeks of antimicrobial therapy may not be required for patients with AC, even if their blood cultures are positive for GPC. In clinical practice, a survey conducted in Asia revealed that most physicians opt for a 4–5 day course of antimicrobial therapy for AC with GPC bacteremia.^5^ However, special consideration is necessary for *Staphylococcus aureus* in the GPC.^53^ If blood cultures are positive for Staphylococcus aureus, antibiotic treatment should be continued for >14 days after confirmation of blood culture negativity.^53^

### 3.3 Fever

Body temperature is an important indicator of AC improvement.^9^ However, RCTs on intra‐abdominal infections have shown that, when the infection source is adequately controlled, the duration of antimicrobial therapy can be shorter than the standard duration, even without clinical and laboratory evidence of infection improvement.^54^ To the best of our knowledge, there is only one retrospective study on the association between AC and fever.^42^ This study employed inverse probability of treatment weighting (IPTW) analysis to investigate whether the presence of fever within 24 h prior to the discontinuation of antimicrobial therapy influences the outcomes of AC. According to this study, the patient's fever status within 24 h before the completion of antimicrobial treatment did not affect the outcomes of cholangitis.

These findings can be interpreted based on several factors. Recent experimental data suggest that the persistence of systemic inflammatory response syndrome may reflect the immune activity of the host rather than the presence of viable bacteria.^55^, ^56^ Thus, a febrile state does not necessarily indicate a prolonged infection, contributing to a growing body of research questioning the reliance on fever‐based medicine.

In patients with AC who present with fever, it is challenging to clinically distinguish whether an infection is ongoing or if the host immune response continues even after the infection has resolved.^57^ In this context, the use of early warning scores such as the national early warning score (NEWS) may be beneficial in assessing the risk of severe outcomes in febrile patients.^58^, ^59^ It is noteworthy that most patients in a previously mentioned retrospective study had a NEWS of 4 or less at the conclusion of antimicrobial therapy, thereby indicating a reduced risk of severity at the time of treatment completion.^42^ This suggests that even if patients are febrile before the completion of antimicrobial therapy, it may not necessarily lead to worse outcomes and may be associated with lower NEWS values.

### 3.4 Hilar multiple biliary obstruction due to malignancy

Multiple hilar biliary obstructions may affect AC outcomes. Bismuth IV obstruction (Figure S1) has been reported to have a higher clinical failure rate for bile duct drainage than other hilar obstruction levels.^60^ Moreover, in patients with hilar biliary obstruction, the number of patients who required re‐drainage of the bile duct was higher in those treated with bismuth‐II (91.9%), bismuth‐IIIa (65.7%), and bismuth‐IV (92.9%) than in those treated with bismuth‐I (22.2%).^61^ However, to the best of our knowledge, only one descriptive retrospective study has reported that AC due to multiple hilar biliary obstructions is associated with worse outcomes than AC due to simple obstruction in the extrahepatic bile duct.^41^ According to this report, in AC cases, the clinical cure rates and 3‐month recurrence rates differ based on the cause of biliary obstruction. For cases of bile duct stones or benign obstruction, the clinical cure rate was 98.1%, and the 3‐month recurrence rate was 3.4%. For simple biliary obstruction due to malignancy (bismuth‐I), the rates were 92.0% and 13.3%. However, in cases of multiple hilar biliary obstruction due to malignancy (Bismuth II–IV), the clinical cure rate was lower (87.1%) and the 3‐month recurrence rate was higher (32.3%). This result may be attributed to the difficulty of achieving complete drainage in multiple hilar biliary obstruction.^61^ Therefore, future research must examine the duration of antimicrobial therapy in AC cases of multiple hilar biliary obstruction due to malignancy (Bismuth II–IV) where drainage is sufficient. Thus, due to the paucity of evidence, the TG18 guidelines regarding this condition do not distinctly differentiate between the treatment strategies for AC arising from hilar multiple biliary obstructions and those for AC resulting from simple extrahepatic obstructions.^5^

In light of previous reports, it is suggested that cases with positive blood cultures, fever, or severe cases excluding those requiring ICU admission may be amenable to standard or short‐term treatment periods. However, in standard‐ or short‐term treatment periods, cases involving multiple hilar biliary obstructions due to malignancy may result in poorer outcomes than simple extrahepatic obstructions, and proper therapeutic approaches may differ between them.

### 3.5 AC complicated by pyogenic liver abscess or acute cholecystitis

A systematic review of the medical literature on pyogenic liver abscesses conducted between 2000 and 2020 revealed that the duration of antibiotic treatment varies significantly.^62^ This review, which included 16 studies encompassing 3933 patients, found that the mean antibiotic treatment duration ranged from 8.4 to 68.9 days, with a pooled mean of 32.7 days. However, this variability in treatment duration did not appear to be influenced by factors such as the age of the participants, type of pathogen, abscess size, or number of abscesses. It is important to note that there is currently no consensus or specific guideline on the optimal duration of antibiotic treatment for pyogenic liver abscess, indicating the need for further randomized controlled trials to determine the best treatment duration for this complex infection. However, RCTs have shown that for intra‐abdominal abscesses where the source of infection is controlled, a median of 4 days (range, 3–5 days) of treatment is sufficient after addressing the source of infection.^54^ Therefore, in cases of pyogenic liver abscess where the infection source is adequately controlled, a shorter duration within the reported range of 8.4 to 68.9 days for antimicrobial therapy may be sufficient.

For mild or moderate acute cholecystitis, antimicrobial therapy for one day has also been reported to be sufficient after successful cholecystectomy.^63^, ^64^ Therefore, for acute cholecystitis with appropriate infection source control, an antibiotic treatment duration of 1–4 days may be sufficient.^54^, ^63^, ^64^

Therefore, while it is difficult to recommend a clear treatment duration for cases of AC complicated by pyogenic liver abscess, in cases of AC complicated by acute cholecystitis where the source of infection is properly controlled, setting an appropriate antibiotic treatment duration for AC should be sufficient for cure.

Table 3 summarizes the proposed duration of antibiotic therapy for each condition.

**TABLE 3:** Antibiotic therapy duration proposed for each specific conditions.

	1–3 days	4–7 days	8 days or longer	Consider extension based on the individual course of each case
Severe	Retrospective studies^6^, ^7^, ^21^, ^46^	TG18, RCT^44^		
Positive blood culture	Retrospective studies^8^, ^21^, ^40^	TG18 (GNR)	TG18 (GPC)	
Fever	Retrospective study^42^			
Hilar multiple biliary obstruction				Retrospective studies^41^, ^60^, ^61^
Complicated by pyogenic liver abscess			Retrospective study^62^	Retrospective study^62^
Complicated by acute cholecystitis	RCT studies^54^, ^63^, ^64^	RCT^54^		

*Note*: The TG18 recommendations for the duration of antimicrobial therapy are based on a conglomeration of diverse evidence integrated through expert opinion, with insufficient specific evidence to support them.

Abbreviations: GNR, gram‐negative rods; GPC, gram‐positive cocci; RCT, randomized controlled trial.

## 4 DURATION OF ANTIBIOTIC ADMINISTRATION IN CASES WHERE RESISTANT BACTERIA TO THE ADMINISTERED ANTIBIOTICS ARE DETECTED

As a general principle in infectious diseases, when bacteria identified from blood cultures show resistance to administered antibiotics, it is recommended to switch to a susceptible antibiotic and reset the treatment duration.^65^ It has also been reported that the outcomes of AC worsen when bacteria identified from blood cultures are resistant to antibiotics.^66^ However, a critical issue with these reports were that they did not include factors that affect the outcome of cholangitis in the multivariate analysis, such as severity, age, time from consultation to ERCP, ERCP success rate, or antibiotic treatment duration. Conversely, there are reports suggesting that if resistance to administered antibiotics is found in bacteria identified from blood or bile cultures, changing the antibiotic is not necessary if the biliary drainage is successful.^8^, ^45^, ^67^ Furthermore, prospective studies indicated that infection with antibiotic‐resistant bacteria is not a predictor of mortality in critically ill patients.^68^ Therefore, although no phase 3 comparative trials have verified the appropriateness of discontinuing antibiotics for AC patients with antibiotic‐resistant bacteria, it may be possible to conclude antibiotic administration without changing the medication if AC shows improvement when antibiotic resistance is identified.

## 5 ASSOCIATION BETWEEN ANTIBIOTIC DURATION AND ADVERSE EVENTS

The greatest disadvantage of long‐term antibiotic administration is the development of antibiotic‐resistant bacteria.^11^, ^35^, ^36^, ^69^, ^70^ Another study reported that the hazard ratio for the emergence of resistant organisms increased by 1.04 for every additional day of exposure to β‐lactam antibiotics.^11^

Long‐term antibiotic administration can lead to several adverse outcomes in addition to the emergence of resistant bacteria. These include an increase in *Clostridium difficile* (CD) colitis and invasive candidiasis, a rise in organ damage caused by the antibiotics themselves, increased healthcare costs, and in some infections (such as intra‐abdominal infections), a reported increase in mortality rates.^12^, ^13^, ^14^, ^15^, ^16^ Additionally, several adverse events (such as liver damage, kidney damage, drug‐induced enteritis, CD colitis, and fever) have been reported to depend on the duration of administration. With each additional day of antibiotic administration, the risk of adverse events increased by 4%.^71^ Shortening the duration of antibiotic administration is expected to reduce the emergence of resistant bacteria, adverse events, the length of hospital stay, and healthcare costs.^11^, ^71^, ^72^

## 6 SELECTION OF ANTIBIOTIC TYPES

Antimicrobials might be discontinued before the susceptibility results are known if the trend towards short‐term administration continues. Even if bacteria resistant to the administered antibiotics are detected in cultures, reports suggest that the outcome of AC does not worsen if appropriate biliary drainage is successful.^45^, ^67^ However, it is advisable to select antibiotics based on susceptibility.^5^ From this point of view, important factors for choosing antibiotics with susceptibility include local antibiograms. In addition, TG18 emphasizes that disease severity and the risk of harboring resistant bacteria (especially in cases of healthcare‐associated biliary infections) are important factors in antibiotic selection.^5^

### 6.1 Severity

Cases with greater severity have a higher mortality rate,^3^ and TG18 recommends broad‐spectrum antibiotics for the safe treatment of severe cases safely.^5^ In TG18, *Pseudomonas* and *Enterococcus* spp. were noted as bacteria that required careful coverage in severe cases. For patients with a history of choledochojejunostomy, coverage of the *Bacteroides fragilis* group should be considered. Furthermore, de‐escalation should be performed after the susceptibility results of the causative bacteria are known. To select the most appropriate antibiotic, it is important to constantly update the local antibiogram with the latest information, including data on these bacteria.

### 6.2 Healthcare‐associated biliary infections

In the context of healthcare‐associated biliary infections, TG18 recommends the use of antibiotics similar to those recommended for severe AC cases.^5^ Compared to community‐acquired biliary infections, where *Escherichia coli* is predominantly observed, healthcare‐associated biliary infections have reported similar proportions of *Escherichia coli*, *Klebsiella*, *Pseudomonas*, and *Enterococcus*.^73^ Consequently, TG18 emphasizes the importance of covering *Pseudomonas* in cases of healthcare‐associated biliary infections and has recently highlighted the necessity of vigilance against ESBL‐producing Gram‐negative bacteria. TG18 also suggests coverage for Methicillin‐resistant *Staphylococcus aureus* (MRSA) and *Enterococcus* in cases where colonization is confirmed, and in cases with a history of choledochojejunostomy, it is recommended to cover the *Bacteroides fragilis* group, as in severe cases.

Generally, Healthcare‐Associated Infections (HAIs) are ‘infections that patients get while or soon after receiving health care’.^74^ Specifically, HAIs are defined with treatments such as antibiotics or surgery, use of medical devices, residence in a nursing home or extended care facility, and immunosuppression.^24^, ^75^ In healthcare‐associated pneumonia and healthcare‐associated urinary tract infections, a wide variety of causative organisms—including many resistant bacteria—have been reported, and recommendations including broad‐spectrum antibiotics similar to those suggested by TG18 for healthcare‐associated cholangitis have been presented.^5^, ^24^, ^76^, ^77^, ^78^ However, TG18 does not specifically define healthcare‐associated acute cholangitis, and there is a lack of evidence regarding this condition.^5^ Therefore, to select the most appropriate antibiotics in cases of “Healthcare‐associated biliary infections,” it is crucial to conduct new studies on the causative bacteria of these infections and continually update the local antibiogram specific to them.^5^, ^24^, ^76^, ^77^, ^78^

### 6.3 Local antibiograms

Monitoring and updating local antibiograms are critical for the timely selection of susceptible antibiotics in clinical settings. If antibiotic resistance occurs in more than 20% of local antibiograms, the antibiotic is not suitable as a first empiric therapy.^79^ TG18 recommends that microbiology laboratories report resistance data according to the site of infection and include biliary infections along with other intra‐abdominal infections.^5^

**FIGURE 1:**
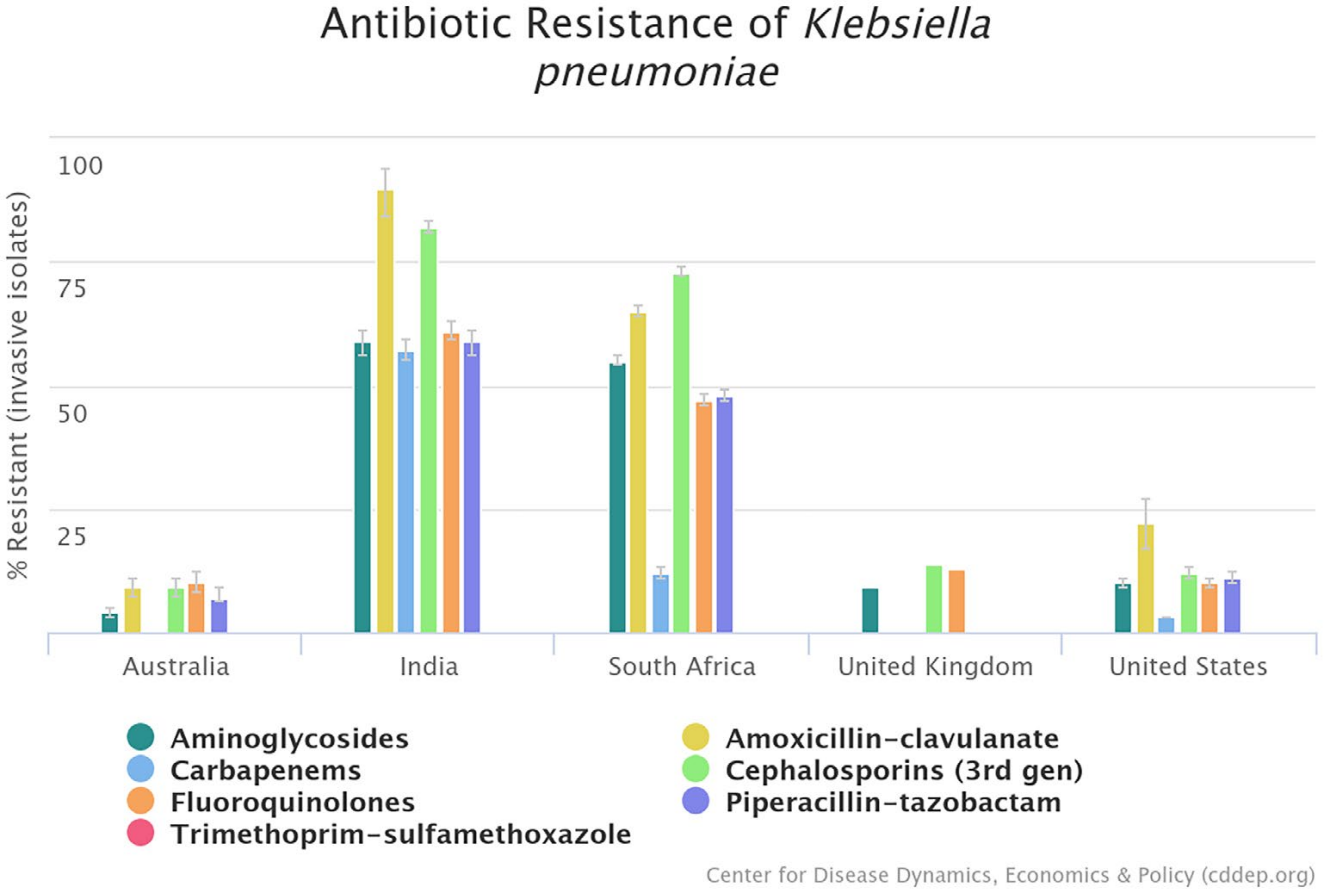
Antibiotic resistance of *Klebsiella pneumonia* in selected countries.

It has been reported that local antibiograms can vary significantly depending on the country or region.^80^ Another study presented data on the global resistance rates of *Klebsiella pneumoniae*, a common causative agent of infectious diseases^18^, ^81^ (Figure 1). Additionally, other papers present national data on AC, a condition often caused by bacteria that tend to show resistance to multiple antibiotics^8^, ^19^, ^20^, ^21^, ^22^ (Table 4). However, caution should be exercised when interpreting this table. The definition of the denominator used to calculate the percentages varied between studies. For example, in reports from Japan, the denominator was the number of cases with positive blood cultures, whereas other reports used the total number of bacteria detected as the denominator. Consequently, a straightforward comparison was not possible. Nevertheless, the situation regarding resistant bacteria is similar in Japan, the United States, and China. Additionally, it can be inferred that the Netherlands has fewer bacterial species, such as *Enterococcus*, *Pseudomonas*, and *Enterobacter*, that often show resistance to multiple drugs.

**TABLE 4:** National data for representative resistant bacteria on acute cholangitis.

	Japan^8^ (*n* = 136)	USA^19^ (*n* = 174)	Germany^20^ (*n* = 78)	Netherlands^21^ (*n* = 187)	China^22^ (*n* = 140)
*Enterococcus* spp.	8.8%	8.6%	20.5%	4.4%	17.2%
*E. coli*	46.3%	28.2%	32.1%	67.5%	45.7%
*Klebsiella* spp.	25.7%	20.7%	14.1%	24.4%	17.7%
*Serratia* spp.					
*Pseudomonas* spp.	2.2%		7.7%		5.1%
*Acinetobacter* spp.					
*Citrobacter* spp.	2.9%		2.6%		0.0%
*Enterobacter* spp.	6.6%		5.1%	3.8%	6.3%

The COBRA trial, which is mentioned several times in this paper, is currently underway in the Netherlands. This trial is a phase 3 comparative study designed to verify whether ultra‐short‐term administration of antibiotics (1 day) following successful biliary drainage in AC is not inferior to the standard duration of administration (4–7 days) in terms of clinical cure rates. It is anticipated that in this trial, termination of antibiotic therapy often occur without waiting for blood culture test results. The characteristic backdrop of the Netherlands, where resistant bacteria are considered extremely rare, should be noted, as it may enable the conduct of a trial that does not depend on blood culture results. However, there are few countries/regions with a low prevalence of resistant bacteria, such as the Netherlands.^8^, ^18^, ^19^, ^20^, ^21^, ^22^, ^80^, ^81^ Therefore, in addition to the COBRA study, randomized controlled trials with different antibiotic resistance profiles are needed. The BOLT‐P3 study (NCT05750966) is currently ongoing in Japan. Similar to the COBRA study, this study set the clinical cure rate as the primary outcome. However, this differs from the COBRA study in that it is a trial comparing a short‐course group receiving 1–3 days of therapy with a standard‐course group receiving 4–7 days of therapy, allowing for adjustment of treatment duration based on blood culture results. The results of these studies are expected to be reported in the next 2–3 years. Including the RCT from India, the PICO for the three RCTs are summarized in the previously mentioned Table 2.

### 6.4 Risks of harboring resistant bacteria

The risk factors for developing infections caused by antimicrobial drug‐resistant pathogens include a history of antimicrobial exposure.^23^, ^24^ Other risk factors include exposure to medical devices and healthcare workers,^24^, ^25^, ^26^, ^27^, ^28^, ^29^, ^30^ regular use of proton pump inhibitors (PPIs),^31^ implantation of biliary stents,^32^, ^33^, ^38^ and prior endoscopic sphincterotomy (EST).^19^, ^34^, ^38^ Moreover, the long‐term administration of antimicrobial agents, especially in patients with pneumonia, poses a risk for the emergence of multidrug‐resistant bacteria.^11^, ^35^, ^36^, ^37^ A recent report indicated that standard duration of antibiotic administration in patients with AC may contribute to the emergence of antibiotic‐resistant bacteria.^39^ According to this report, the standard duration of antibiotic therapy recommended by the TG18 has been associated with an increased occurrence of resistant bacteria compared to shorter courses of treatment.

The relative weights of each of these factors in terms of their contribution to the risk of the emergence of antibiotic‐resistant bacteria are still unclear.

### 6.5 Selection of antibiotic types for clinical practice

From the discussions so far, it is clear that important information for selecting antibiotics for AC includes understanding the local antibiogram and assessing patient factors, such as severity and risk factors for harboring resistant bacteria. Furthermore, it is critical to consider the results of previous culture tests in the cases where they were conducted.^5^ We present provisional benchmarks for antibiotic selection at our facility based on limited evidence (Table S1).

Antibiotics promote the emergence of resistant bacteria.^23^, ^24^ Furthermore, although a difference in the incidence of resistant bacteria depending on the type of antimicrobial agent used is possible,^11^, ^82^ several comparative studies have found no evidence that antibiotic type contributes to the development of bacterial resistance.^83^ These findings imply that both narrow‐ and broad‐spectrum antibiotics present a similar risk of promoting resistant bacteria, thereby indicating that careful use of broad‐spectrum agents is essential to preserve the efficacy of antimicrobial treatments for future infections.

## 7 CONCLUSION

In terms of effectiveness, the Indian RCT compared 4 and 8 days within the standard treatment duration and demonstrated the noninferiority of a shorter 4‐day duration in patients with common AC after successful biliary drainage. Furthermore, retrospective studies suggested that outcomes did not worsen even after 3 days or less, and this may hold true even in cases with severe grade, positive blood cultures, or fever. However, when bacteria resistant to the administered antibiotics are detected in cultures or in cases of multiple hilar biliary obstructions, it is advisable to determine the duration of therapy while observing the clinical course of each case. There have been reports of fewer emergences of resistant pathogens and fewer adverse events as advantages of short‐term treatment. Two RCTs are currently underway, and their results are anticipated.

While it is challenging to make specific recommendations regarding the type of antibiotics, it is important to consider the local antibiogram, patient factors such as the severity of AC or risk factors for resistant bacteria, and the results of previous culture tests, if available. The most crucial goal is to safely cure patients with AC. However, it is also important to consider the problems that an increase in resistant bacteria can cause in the future.

## AUTHOR CONTRIBUTIONS

Conceptualization: Sakue Masuda and Yoshinori Imamura. Methodology: Sakue Masuda. Software; Sakue Masuda. Validation: Yoshinori Imamura, Kazuya Koizumi, Karen Kimura, Ryuhei Jinushi and Shomei Ryozawa. Investigation: Sakue Masuda. Resources: Sakue Masuda. Writing—original draft preparation, Sakue Masuda. Writing—review and editing, Sakue Masuda. Visualization: Sakue Masuda. Supervision: Yoshinori Imamura, Kazuya Koizumi and Shomei Ryozawa. Project administration, Sakue Masuda. Funding acquisition: Not applicable. Ryuhei Jinushi and Kazuya Koizumi provided input for writing the paper. All the authors have read and agreed to the published version of this manuscript.

## CONFLICT OF INTEREST STATEMENT

The authors have no conflicts of interest to declare.

## Supporting information


**Figure S1.** Illustration of the Bismuth classification for cholangiocarcinoma.

**Table S1.** Benchmarks for antibiotic selection at our facility.
