# Supplementary figures and images for: Navigating antibiotic therapy in acute cholangitis: Best practices and new insights
Source: J Hepatobiliary Pancreat Sci. 2024 Nov 13;32(1):44–57. doi: 10.1002/jhbp.12087 (PMC11780307; doi:10.1002/jhbp.12087)

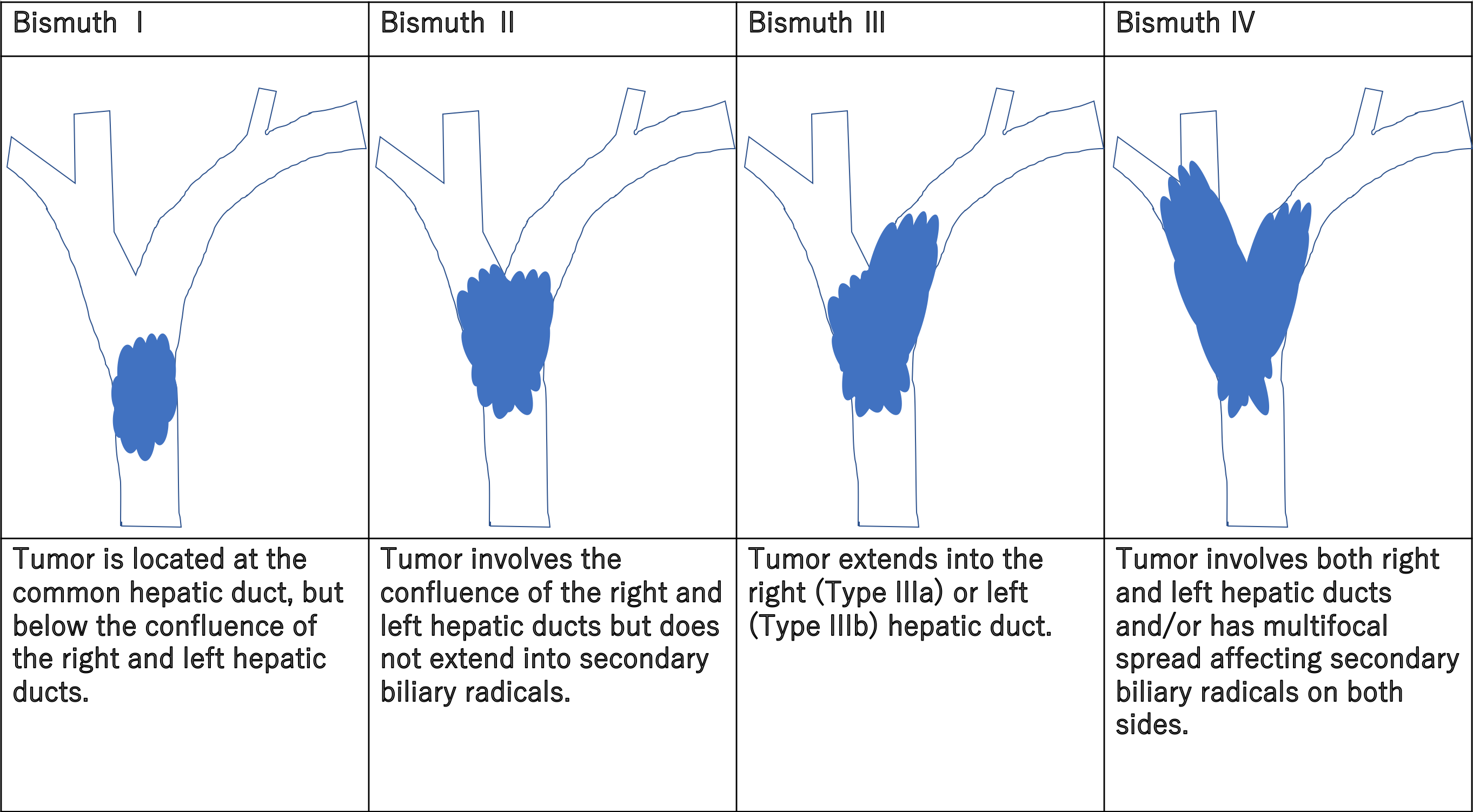

Supplement: Supplementary file 1 — Figure S1. Illustration of the Bismuth classification for cholangiocarcinoma. [file JHBP-32-44-s001.png]
