# Supplementary material for: Navigating antibiotic therapy in acute cholangitis: Best practices and new insights
Source: J Hepatobiliary Pancreat Sci. 2024 Nov 13;32(1):44–57. doi: 10.1002/jhbp.12087 (PMC11780307; doi:10.1002/jhbp.12087)
Supplement: Supplementary file 2 — Table S1. Benchmarks for antibiotic selection at our facility. [file JHBP-32-44-s002.docx]

**Supplementary Table 1.** Benchmarks for antibiotic selection at our facility

|  | Mile | Moderate | Severe (not requires intensive care) | Severe (requires intensive care) |
| --- | --- | --- | --- | --- |
| Risk for resistant bacteria | Narrow | Narrow/Broad | Broad | Broad |
| No risk for resistant bacteria | Narrow | Narrow | Narrow/Broad | Broad |
